# Supplementary material for: FGFR inhibition blocks NF-ĸB-dependent glucose metabolism and confers metabolic vulnerabilities in cholangiocarcinoma
Source: Nat Commun. 2024 May 7;15:3805. doi: 10.1038/s41467-024-47514-y (PMC11076599; doi:10.1038/s41467-024-47514-y)
Supplement: Supplementary file 1 — Supplementary Information [file 41467_2024_47514_MOESM1_ESM.pdf]

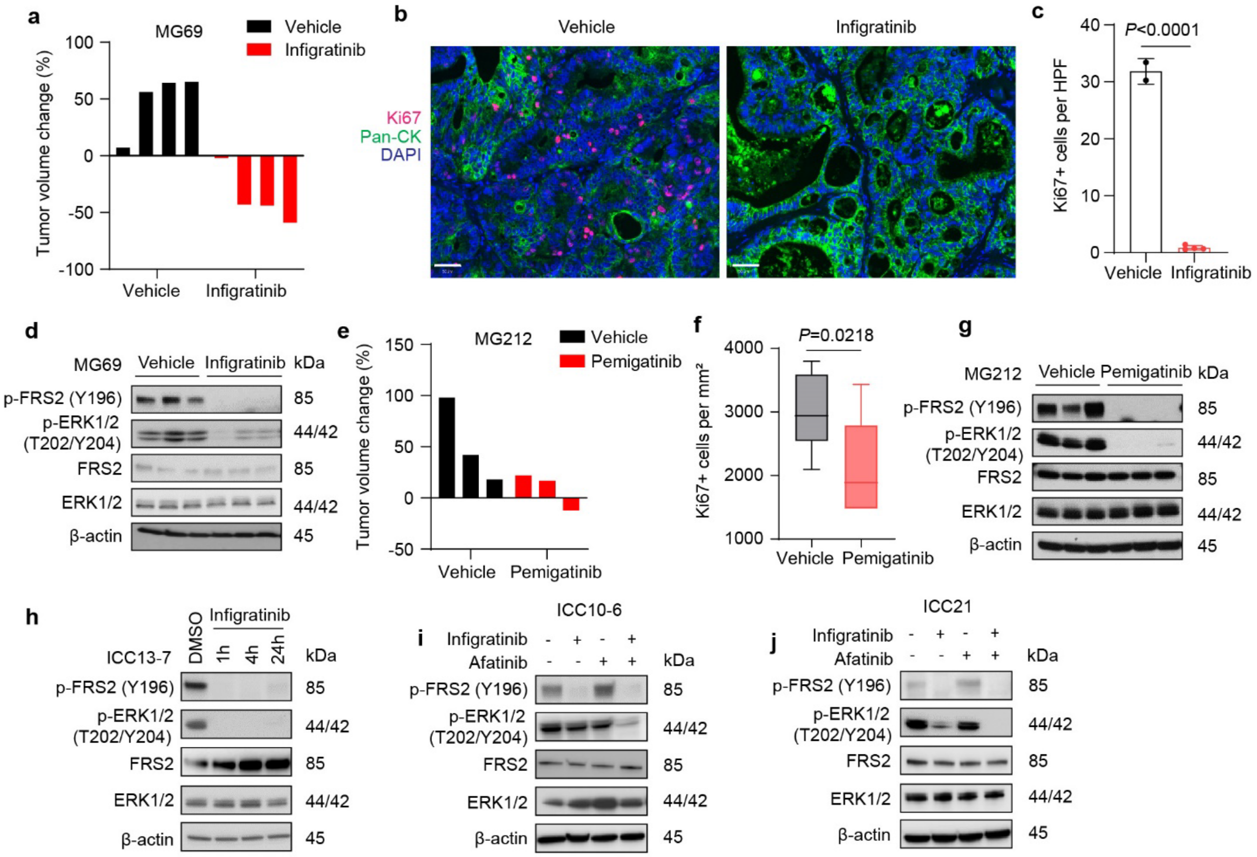


**Supplementary Figure 1. Characterization of FGFR2-fusion+ ICC models.** (**a-d**) The MG69 PDX model was treated with 15 mg per kg infigratinib (n=4) or vehicle (n=4) for 10 days. **a** Waterfall plot showing tumor volume changes. **b-c** Representative immunofluorescence staining for Ki67 (magenta), Pan-CK (green) and DAPI (blue) (**b**), and quantification of the staining data (n=2 mice for vehicle; n=4 mice for infigratinib) (**c**). **d** Immunoblot analysis of the indicated signaling proteins in tumor lysates (n=3 mice). (**e-g**) The MG212 PDX model was treated with 1 mg per kg pemigatinib (n=3) or vehicle (n=3) for 11 days. **e** Waterfall plot showing tumor volume changes. **f** Quantification of Ki67 staining. The box plot shows the center line as the median, and the whiskers' boundary represents the minimum and maximum values of the dataset. The box extends from the 25th to 75th percentiles. **g** Immunoblot analysis of indicated signaling proteins in tumor lysates. **h-j** Immunoblot analysis of indicated signaling proteins in ICC13-7 cells treated with 100 nM infigratinib for indicated time points (**h**), and in ICC10-6 (**i**) and ICC21 cells (**j**) treated

with single agent 100 nM infigratinib, 100 nM afatinib, or the combination, for 24 hours. Data represent means ± SD. Student's t-test (two-tailed) was performed. Western blots were repeated three times. Source data are provided as a Source Data file.

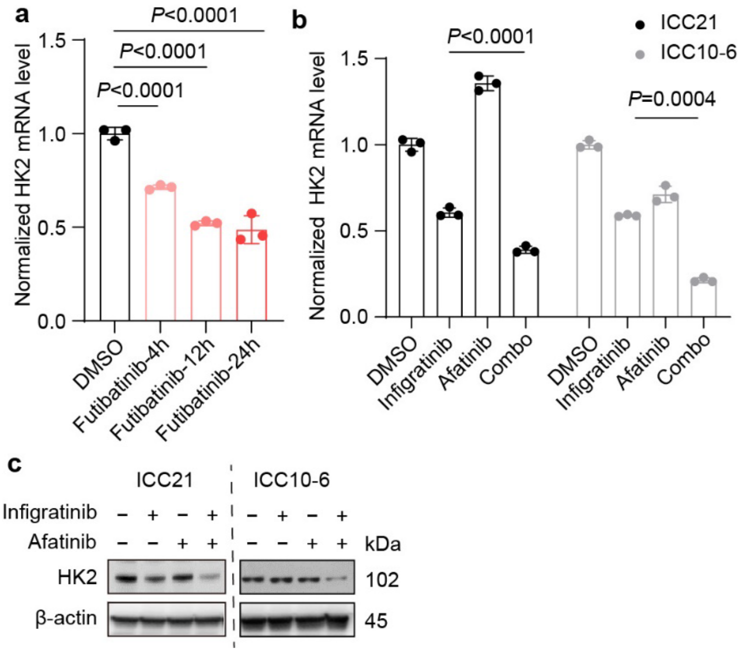


**Supplementary Figure 2. FGFR signaling is required to sustain glycolytic gene expression in FGFR2-fusion+ ICC. a** Relative HK2 mRNA expression in ICC13-7 cells treated with 75 nM futibatinib for the indicated time points (n=3 biological replicates). **b** Relative HK2 mRNA expression in ICC21 and ICC10-6 cells treated with DMSO, single agent infigratinib 100 nM, afatinib 100 nM, or the combination for 4 hours (n=3 biological replicates). **c** Immunoblot of the indicated proteins in ICC21 and ICC10-6 cells treated with DMSO, single agent infigratinib 100 nM, afatinib 100 nM, or the combination for 24 hours. It was repeated three times. Data represent means ± SD. One-way ANOVA multiple comparisons were performed. Source data are provided as a Source Data file.

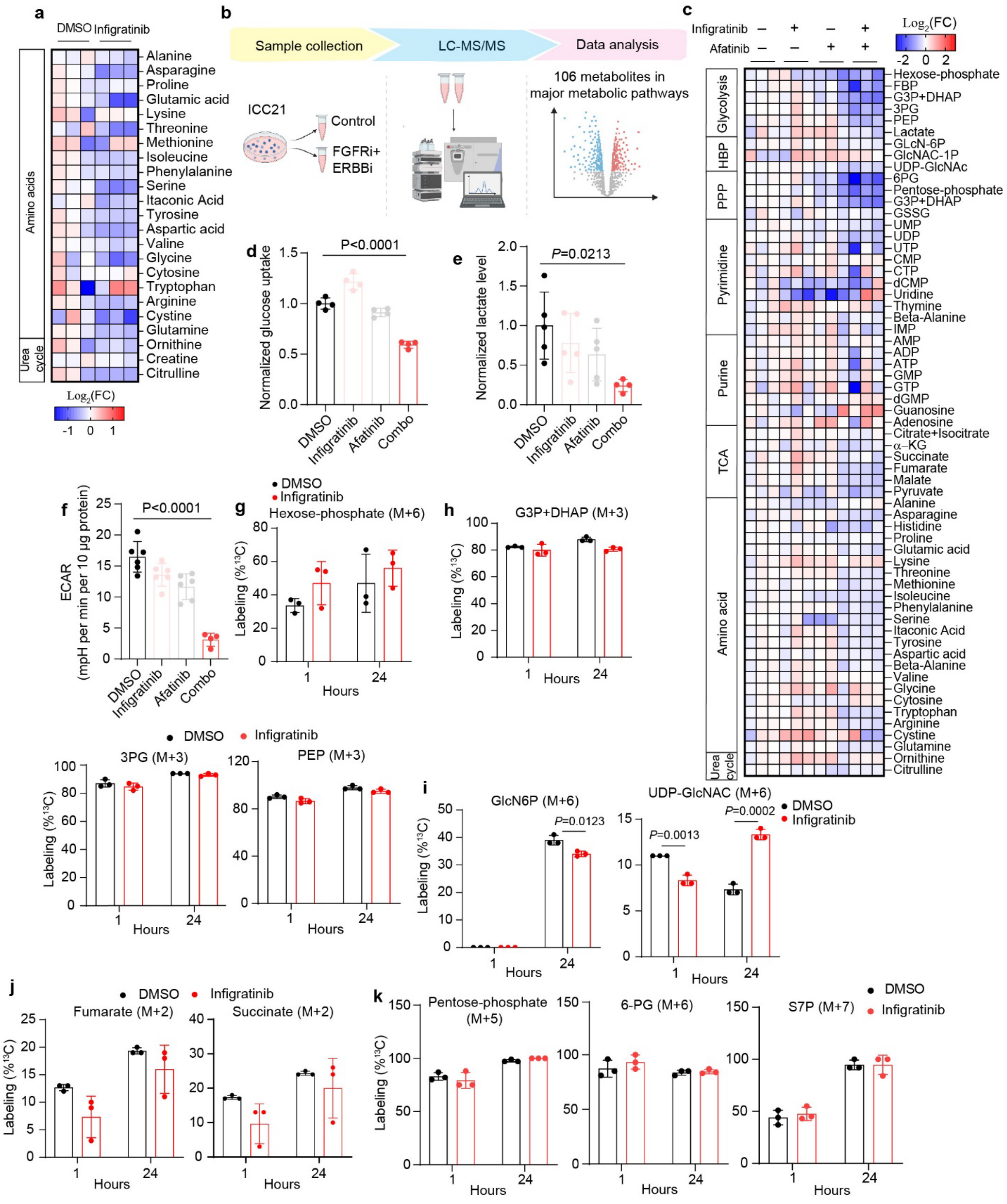


**Supplementary Figure 3. FGFR2-signaling maintains hyperactive glucose metabolism in FGFR2-fusion+ ICC. a** Heatmap depicting changes amino acids and urea cycle metabolites in

ICC13-7 cells treated with 100 nM infigratinib or DMSO for 24 hours. Data are normalized to the DMSO condition and presented as $\mathrm{Log}_2$ transformation (n=3 biological replicates). **b** Schematic of workflow. Created with BioRender.com. **c** Heatmap depicting metabolite changes from different pathways in ICC21 cells treated with DMSO, single agent infigratinib 100 nM, afatinib 100 nM, or the combination for 24 hours. Data are normalized to the DMSO condition and presented as $\mathrm{Log}_2$ transformation (n=3 biological replicates). **d-f** Relative changes of glucose uptake (n=4 biological replicates) (**d**), lactate level (n=5 samples for DMSO/infigratinib/afatinib, n=4 samples for combo) (**e**), and extracellular acid rate (ECAR) (n=6 samples for DMSO/infigratinib/afatinib, n=4 samples for combo) (**f**) in ICC21 cells treated with DMSO, single agent infigratinib 100 nM, afatinib 100 nM, or the combination for 24 hours. **g-k** $^{13}$C enrichment of metabolites after U-$^{13}C_6$-glucose labeling for 1 or 24 hours in ICC13-7 cells, which were treated with 100 nM infigratinib or DMSO for 24 hours before labeling (n=3 biological replicates). Data represent means ± SD. One-way ANOVA multiple comparisons were performed for **d-f**. Student’s t-tests (two-tailed) were performed for **g-k**. Source data are provided as a Source Data file.

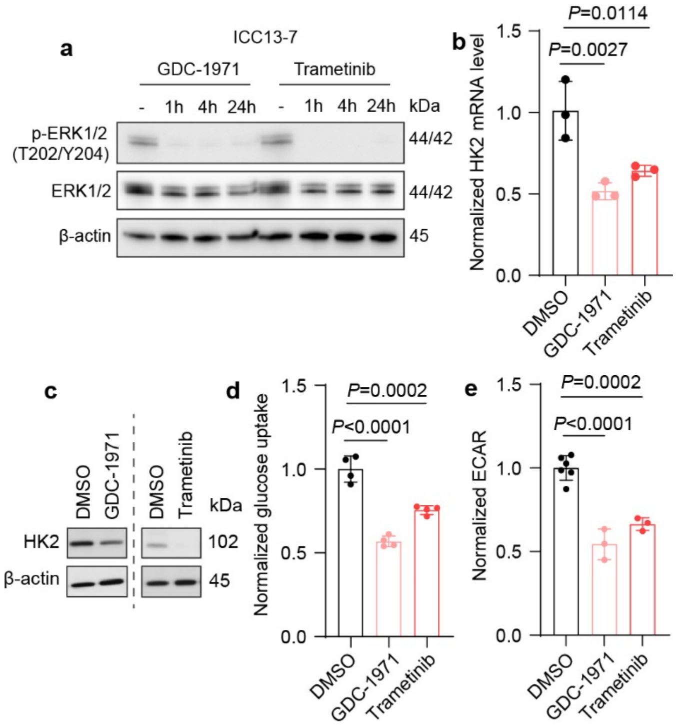


**Supplementary Figure 4. FGFR2 regulates glucose metabolism via MAPK signaling in FGFR2-fusion+ ICC. a** Immunoblot analysis showing the expected signaling changes in ICC13-7 cells treated with 100 nM SHP2 inhibitor, GDC-1971, or 100 nM MEK inhibitor, trametinib, for the indicated time points. **b-c** Relative HK2 mRNA expression (n=3 biological replicates) (**b**) and protein levels (**c**) in ICC13-7 cells treated with 100 nM GDC-1971 or 100 nM trametinib for 24 hours. **d-e** Relative changes of glucose uptake (n=4 biological replicates) (**d**) and ECAR (n=6 samples for DMSO, n=3 samples for RLY-1971/Trametinib) (**e**) in ICC13-7 cells treated with 100 nM GDC-1971 or 100 nM trametinib for 24 hours. For bar graphs, data represent means ± SD. One-way ANOVA multiple comparisons were performed. The Western blots were repeated three times. Source data are provided as a Source Data file.

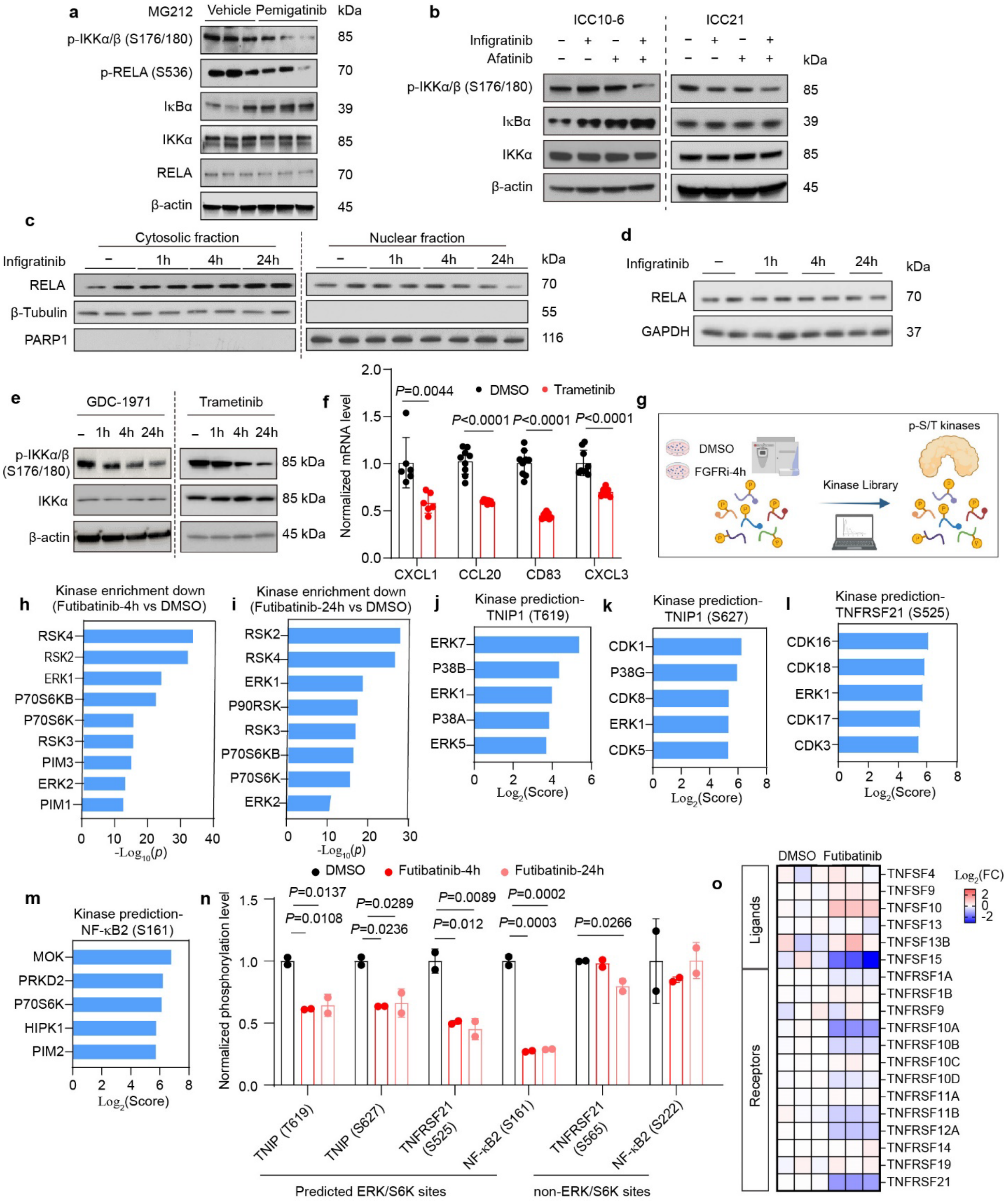

**Supplementary Figure 5. FGFR signaling activates the NF-κB pathway in FGFR2-fusion+ ICC. a-b** Immunoblot analysis of the indicated NF-κB signaling proteins in PDX MG212 treated

with vehicle or pemigatinib (1 mg per kg) for 11 days (n=3 mice each group) (**a**), and in ICC10-6 and ICC21 cells treated with DMSO, 100 nM infigratinib, 100 nM afatinib, or the combination for 24 hours (**b**). **c-d** Immunoblot analysis of RELA levels in cytosolic/nuclear fractions (**c**) and whole cell lysates (**d**) from ICC13-7 cells treated with 100 nM infigratinib or DMSO for the indicated time points. **e** Immunoblot analysis of indicated proteins in ICC13-7 cells treated with 100 nM GDC-1971, 100 nM trametinib or DMSO for the indicated times. **f** Relative mRNA levels of established NF-κB targets in ICC13-7 cells treated with 100 nM trametinib or DMSO for 4 hours (n=6 biological replicates for CXCL1, n=9 biological replicates for other groups). **g** Schematic of phosphoproteomics coupled with prediction of Ser/Thr kinase activity. **h-i** Kinase enrichment analysis of pS/T-carrying peptides using the Kinase Library platform predicts the kinases whose activity is most downregulated in ICC13-7 cells in response to 75 nM futibatinib treatment for 4 (**h**) or 24 hours (**i**). The top-ranked pathways are shown. **j-m** Predicted kinases for the indicated phosphorylation events. $Log_2$(Score)>0 is considered favorable kinases for a given site; $Log_2$(Score)<0 indicates unfavorable kinases. Top 5 ranked-favorable kinases are listed. ERK or P70S6K was labeled bold. **n** Normalized phosphorylation level of NF-κB components with predicted ERK/S6K and non-ERK/S6K sites (n=2 biological replicates). **o** Heatmap of relative mRNA level of NF-κB ligands and receptors in ICC13-7 cell line treated with 75 nM futibatinib or DMSO for 4 hours (n=3 biological replicates). Data represent means ± SD. **f**: Student's t-test (two-tailed). **n**: one-way ANOVA multiple comparisons. Western blots were repeated three times. Source data are provided as a Source Data file.

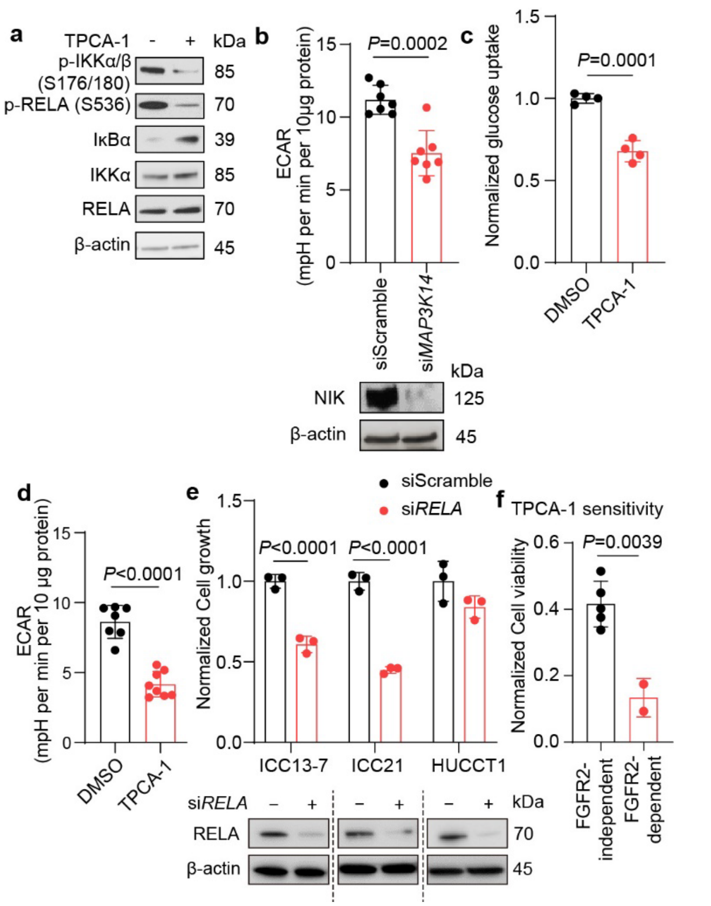


**Supplementary Figure 6. Disruption of the NF-κB pathway dampens glucose metabolism.** **a** Immunoblot analysis of NF-κB signaling components in ICC13-7 cells treated with vehicle or 5 µM TPCA-1 for 24 hours. **b** Relative changes in ECAR normalized to protein amount in ICC13-7 cells transfected with siRNA against MAP3K14 (NIK). The immunoblot analysis on the bottom confirmed knock-down efficiency (n=7 biological replicates). **c-d** Relative changes in glucose uptake (n=4 biological replicates) (**c**), and ECAR (n=7 samples for DMSO, n=8 samples for TPCA-1) (**d**) in ICC21 treated with 5 µM TPCA-1 or DMSO for 24 hours. **e** The growth of cell lines transfected with siRNA against RELA normalized to control (n=3 biological replicates). The immunoblot analysis on the bottom confirmed knock-down efficiency. **f** Relative TPCA-1 sensitivity of FGFR2-dependent cell lines normalized to FGFR2-independent cell lines. Each dot represents one cell line (n=5 samples for FGFR2-independent, including ICC12, ICC10-6, ICC10-

8, CC-SW-1 and SG231; n=2 samples for FGFR2-dependent, including ICC13-7 and ICC21). Data represent means ± SD. Student's t-test (two-tailed) was performed. Western blots were repeated three times. Source data are provided as a Source Data file.

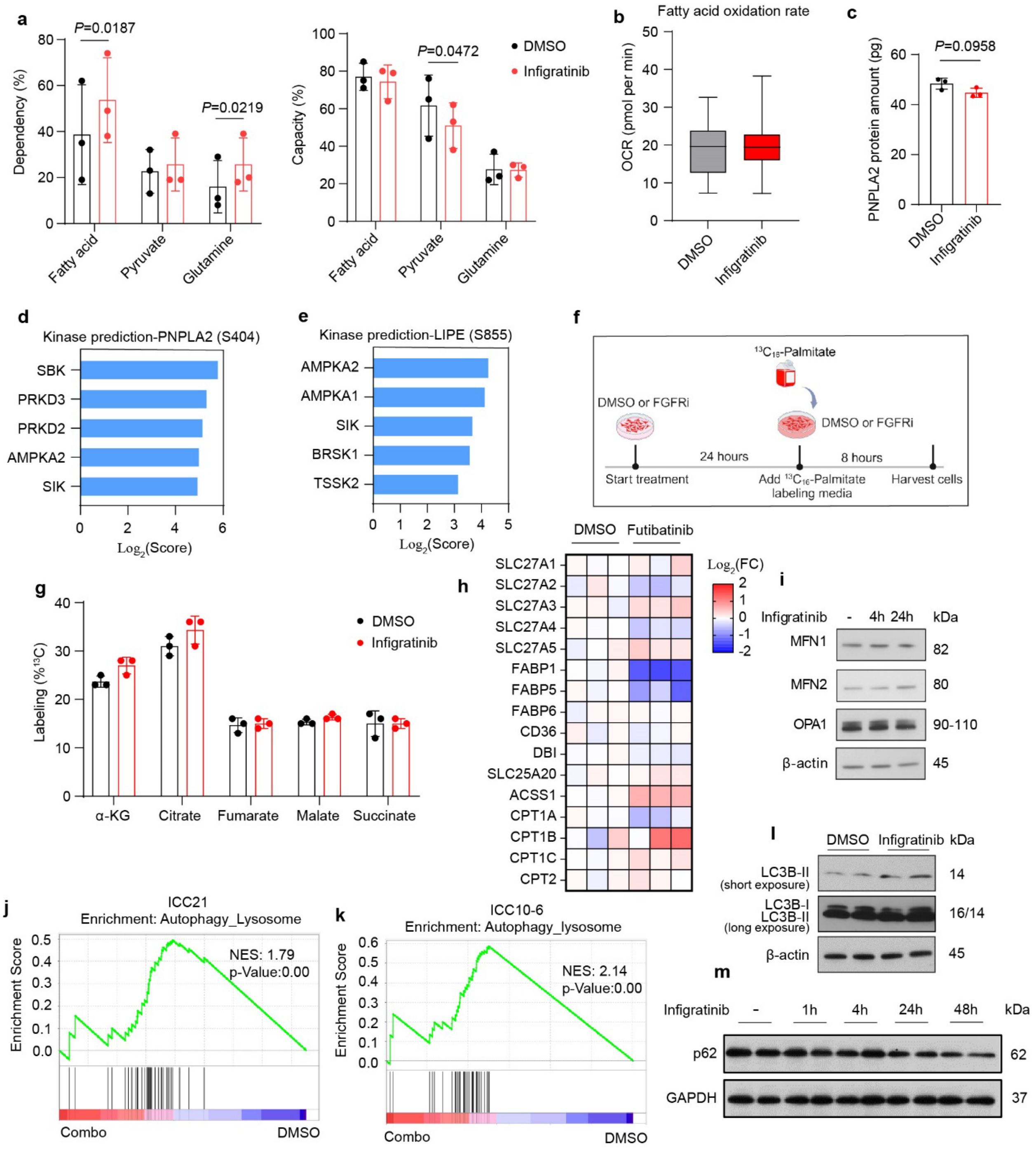


**Supplementary Figure 7. Inhibition of FGFR signaling leads to adaptative changes in mitochondrial fuel source utilization and autophagy-lysosome gene expression. a** Analysis of the dependency and capacity of mitochondria fuels (fatty acid, pyruvate, and glutamine) by Agilent Seahorse XF Mito Fuel Flex Test kit in ICC21 cells treated with 100 nM infigratinib or

DMSO for 24 hours (n=3 biological replicates). **b** Analysis of fatty acid oxidation rate in ICC13-7 cells treated with 100 nM infigratinib or DMSO for 24 hours, calculated by subtracting OCR upon treatment with 4 µM Etomoxir from basal OCR (n=32 samples). Box plots show the center line as the median, and the whiskers' boundary represents the minimum and maximum values of the dataset. The box extends from the 25th to 75th percentiles. **c** PNPLA2 protein levels were measured by ELISA in ICC13-7 cells treated with 100 nM infigratinib for 48 hours (n=3 biological replicates). **d-e** Predicted kinases for the indicated phosphorylation events. AMPKs were labeled bold. **f** Schematic of workflow for the $^{13}C_{16}$-palmitate tracing experiment (n=3 biological replicates). **g** Percentage of $^{13}C$ enrichment for different TCA cycle metabolites (M+2) after $^{13}C_{16}$-palmitate labeling for 8 hours in ICC13-7 cells. Cells were pre-treated with 100 nM infigratinib or DMSO for 24 hours prior to labeling and maintained under these conditions during the labeling step. **h** RNAseq data of the indicated factors in ICC13-7 cells treated with futibatinib (75 nM, 24 hours or DMSO) (n=3 biological replicates). Data were normalized to the vehicle condition and presented as $Log_2$ transformation. **i** Immunoblot analysis of the indicated mito-fusion proteins in ICC13-7 cells treated at 4 hours and 24 hours with infigratinib (100 nM). **j-k** GSEA plots of Autophagy_Lysosome gene signature (1) in ICC21 (**j**) and ICC10-6 (**k**) cells treated with the combination of 100 nM infigratinib and afatinib vs DMSO. **l** Immunoblot analysis of LC3B protein levels in ICC13-7 treated with 100 nM infigratinib for 4 hours and 24 hours. **m** Immunoblot analysis of p62 protein levels in ICC13-7 treated with 100 nM infigratinib for the indicated times. Data represent means ± SD. Student's t-test (two-tailed) was performed. Western blots were repeated three times. Source data are provided as a Source Data file.

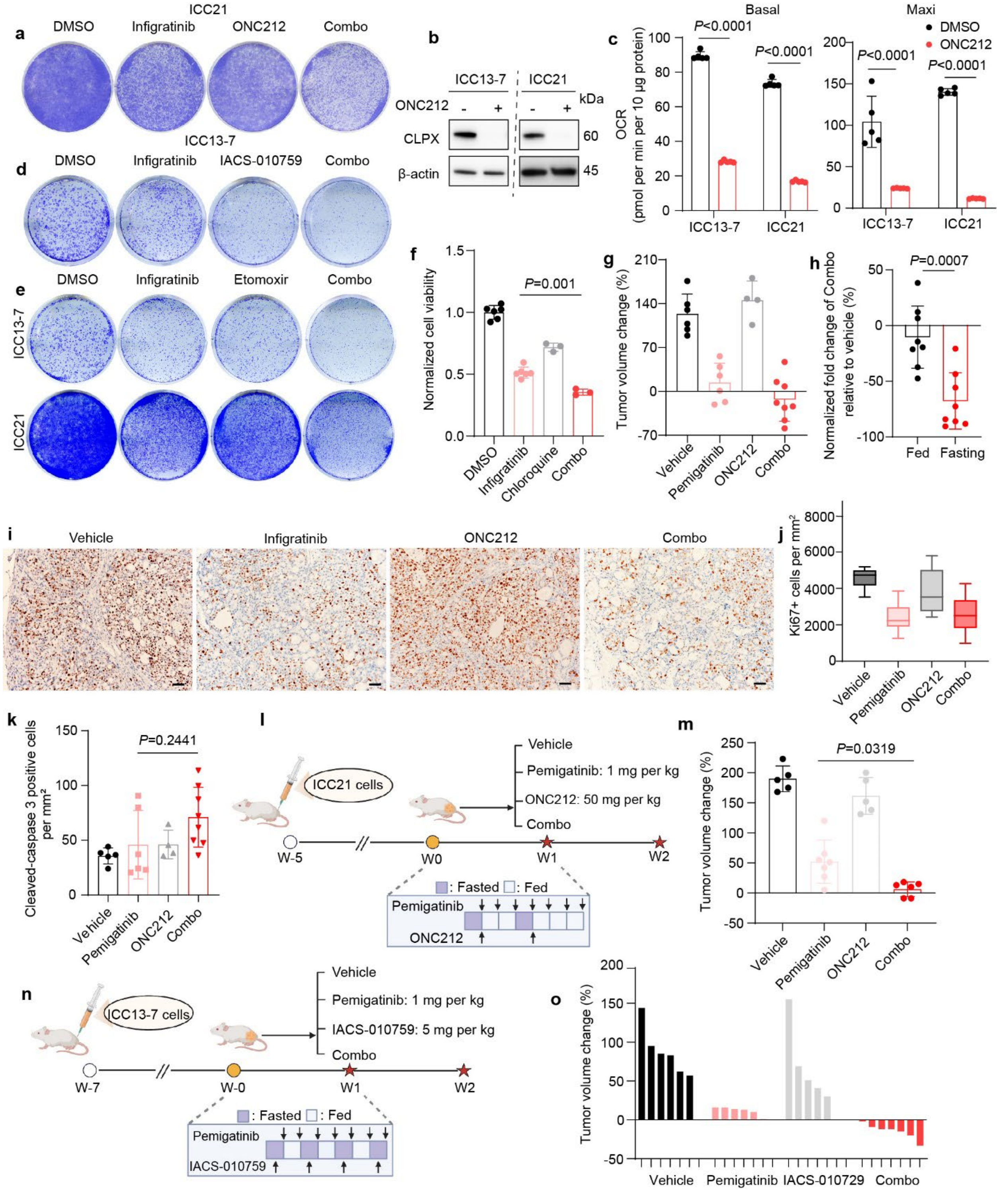


**Supplementary Figure 8. Targeting adaptive metabolic pathways increases FGFRi efficacy against FGFR2 fusion+ ICC.** **a** Cell viability (crystal violet staining) of ICC21 cells treated with

DMSO, infigratinib (50 nM), ONC212 (20 nM), or the combination. **b** Immunoblot of ICC13-7 and ICC21 cells treated with 100 nM ONC212 or DMSO for 48 hours, which was repeated 3 times. **c** Basal and maximal OCR in ICC13-7 and ICC21 cells treated with 100 nM ONC212 or DMSO for 48 hours (n=5 biological replicates). **d-e** Crystal violet staining of ICC13-7 treated with DMSO, 50 nM infigratinib, 1 nM IACS-010759, or the combination (**d**), and ICC13-7/ICC21 treated with DMSO, 50 nM infigratinib, 5 μM etomoxir, or the combination (**e**). Data are representative of three independent experiments. **f** Normalized viability of ICC13-7 cells treated 7 days with DMSO (n=6 samples), infigratinib 50 nM (n=6 samples), chloroquine 10 μM (n=3 samples), or the combination (n=3 samples). **g** Relative tumor volume change of ICC13-7 xenografts treated with vehicle (n=6), 1 mg per kg pemigatinib (n=6), 50 mg per kg ONC212 (n=4), or the combination (n=8) for 17 days. **h** Normalized volume changes of ICC13-7 xenografts upon pemigatinib + ONC212 treatment relative to vehicle, under fed (n=8) and fasting conditions (n=8). **i-j** IHC for Ki67 in ICC13-7 xenografts treated as indicated. **i** Representative images. **j** Quantification. Box plots: center line: median; whiskers' boundary: minimum and maximum values. The box extends from the 25th to 75th percentiles. **k-l** Mice harboring ICC21 xenografts (starting tumor volume ~200 $mm^3$) were treated with vehicle (n=5), 1 mg per kg pemigatinib (n=7), 50 mg per kg ONC212 (n=5), or the combination (n=6) for 14 days under the intermittent fasting regimen. **k** Treatment regimen. **l** Tumor volume changes. **m-n** Mice harboring ICC13-7 xenografts tumors (starting volume ~200 $mm^3$) were treated with vehicle (n=6), pemigatinib 1 mg per kg (n=6), IACS-010759 5 mg per kg (n=5), or the combination (n=7) for 17 days under intermittent fasting. **m** Treatment regimen. **n** Waterfall plot showing tumor volume changes of ICC13-7 xenografts upon the indicated treatments. Data represent means ± SD. One-way ANOVA multiple comparisons were performed except for panel **c** and **h**, calculated using the Student's t-test (two-tailed). Scale bar: 50 μm. For **a**, **d** and **e**, data representative of three independent experiments. **k** and **m** were created with BioRender.com. Source data are provided as a Source Data file.

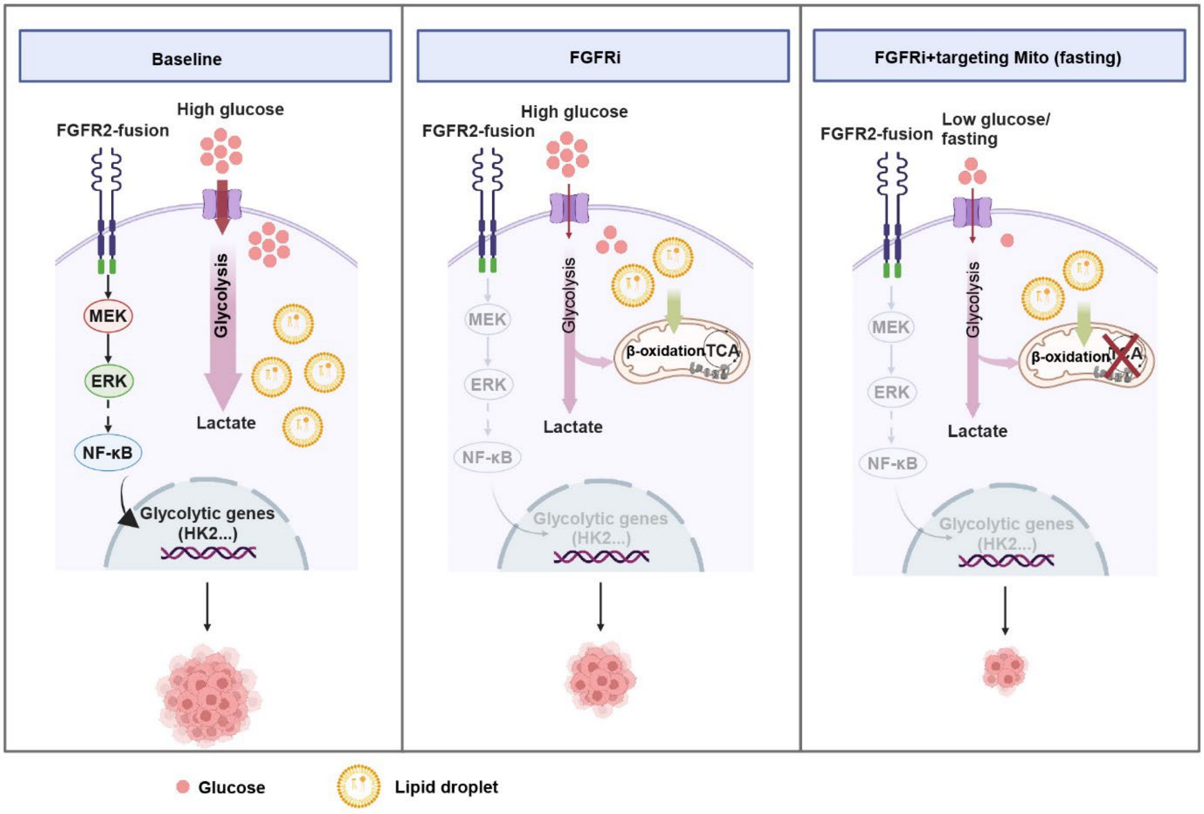


**Supplementary Figure 9. Harnessing adaptive metabolic changes to potentiate FGFRi efficacy in FGFR2-fusion+ ICC.** In FGFR2 fusion+ ICC, activated FGFR2-MEK signaling sustains hyperactive glucose metabolism through NF-κB. Upon FGFR inhibition, glycolytic gene expression and glycolytic activity are dramatically decreased. Switches in fuel source utilization to support mitochondria respiration and sustain cell survival. Targeting mitochondria potentiates FGFR inhibitor efficacy, an effect enhanced in fasting regimen. Created with BioRender.com.

Source Data - Supplementary Figure 1

Fig S1d

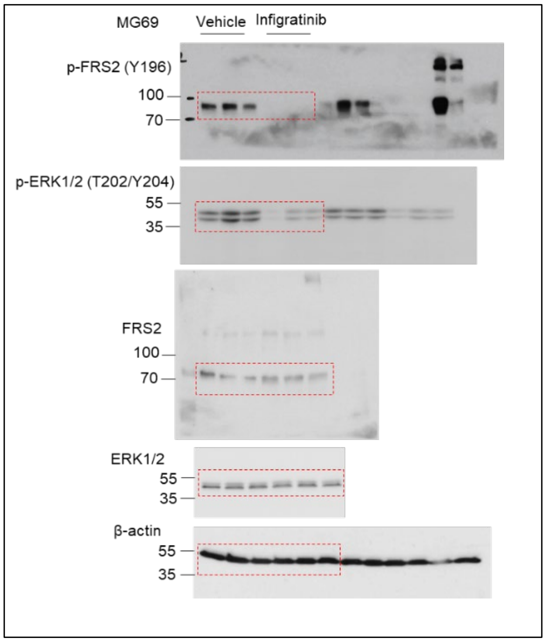


Fig S1g

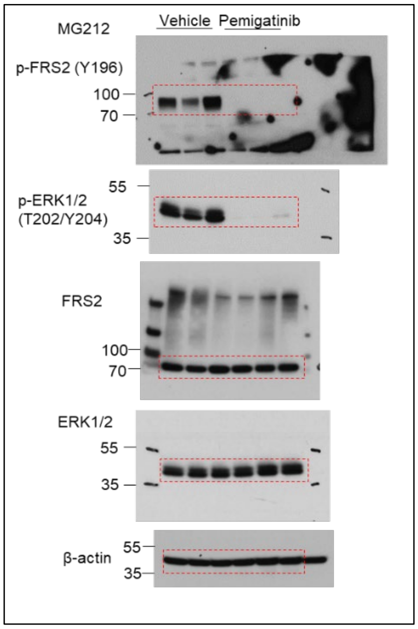


Fig S1h

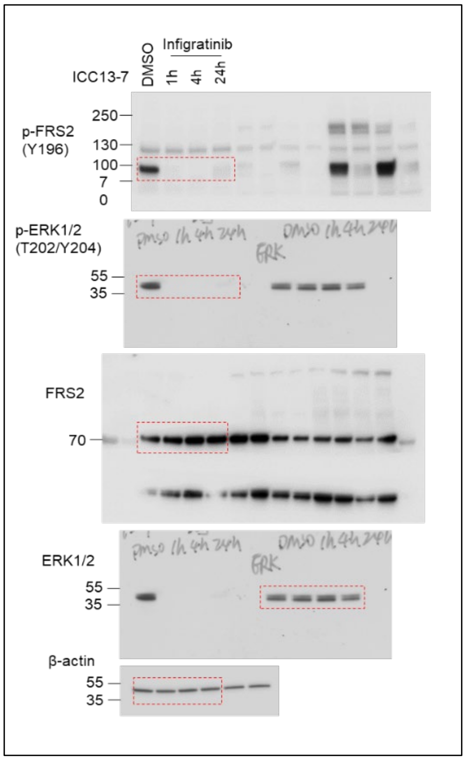


Fig S1i

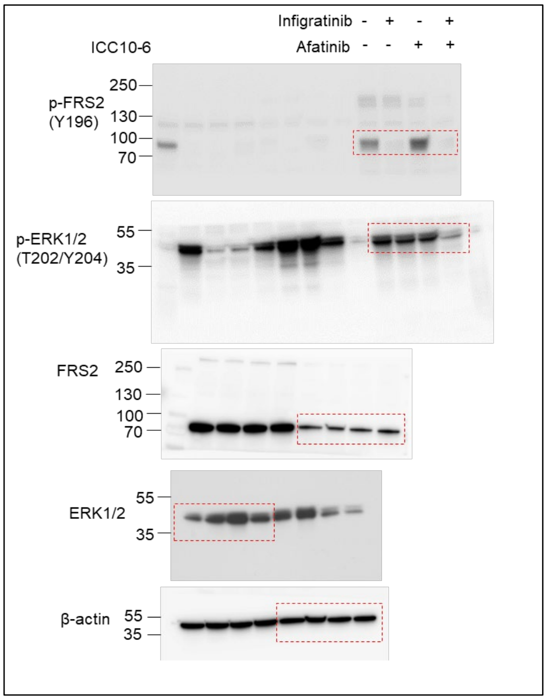

# Source Data - Supplementary Figure 1

Fig S1j

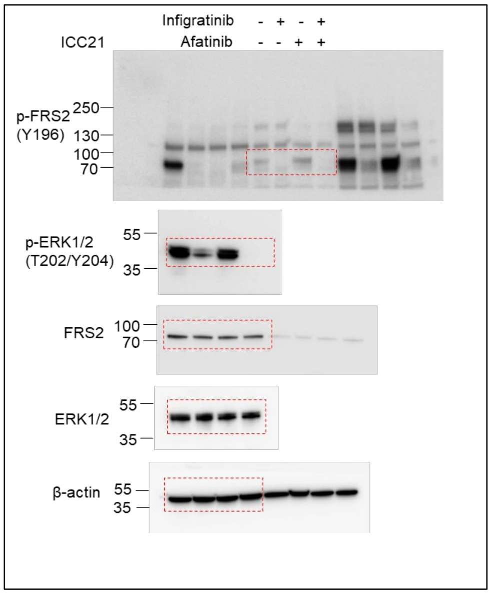

Source Data - Supplementary Figure 2

Fig S2c

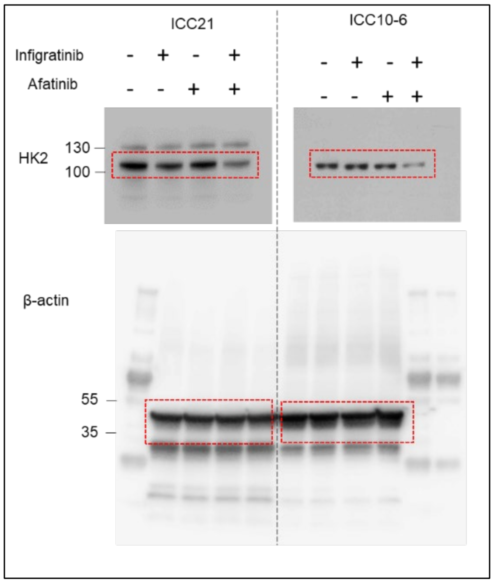

# Source Data - Supplementary Figure 4

Fig S4a

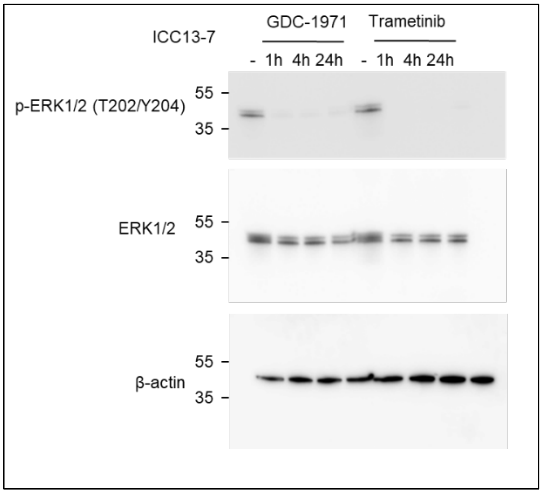


Fig S4c

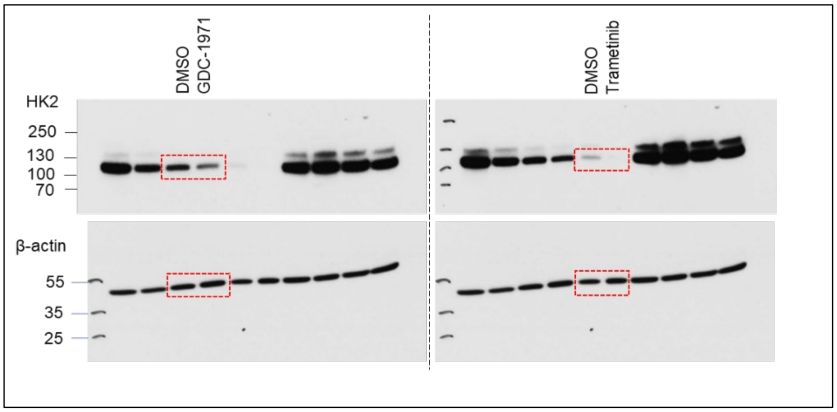

## Source Data - Supplementary Figure 5

Fig S5a

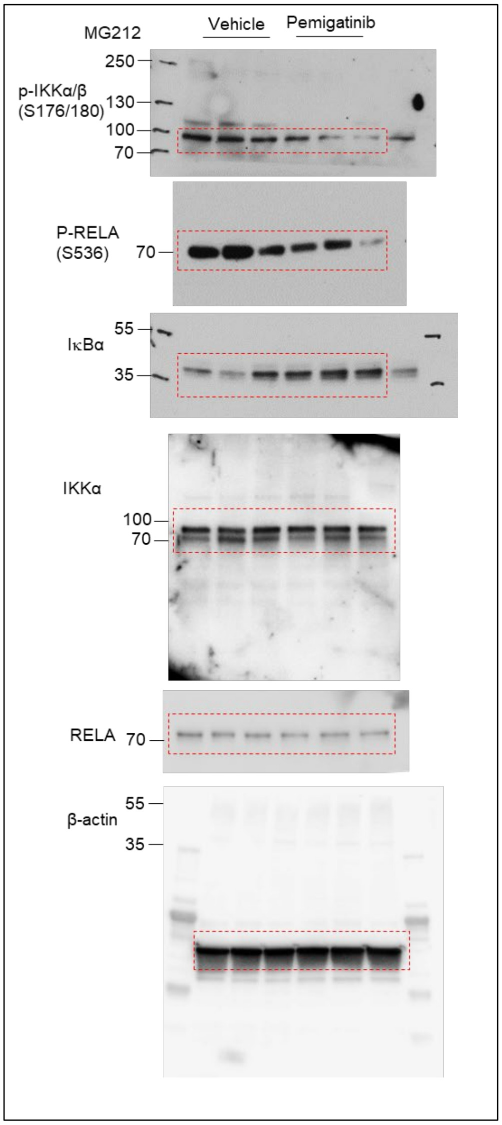


Fig S5b

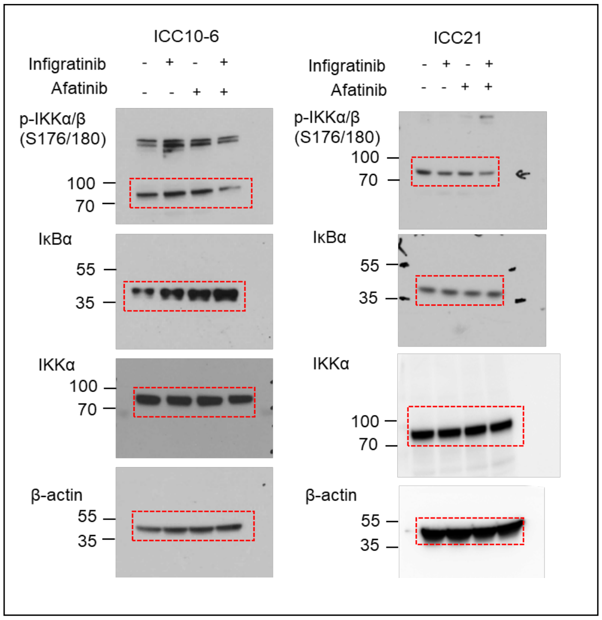


Fig S5c

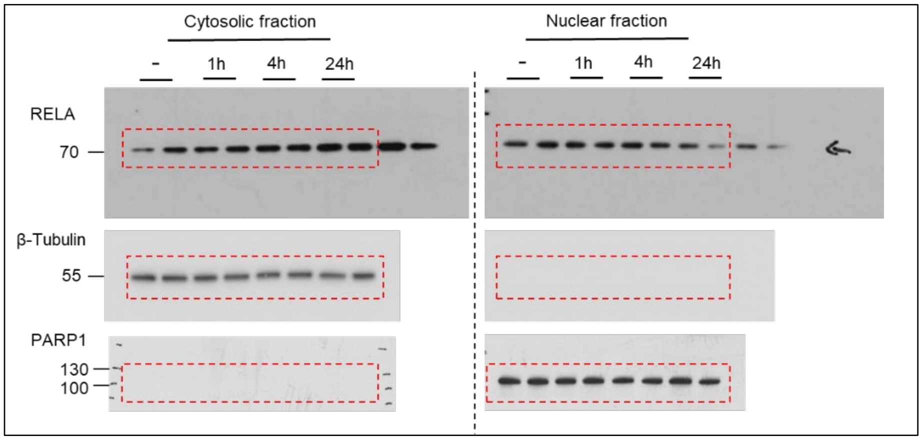

# Source Data - Supplementary Figure 5

Fig S5d

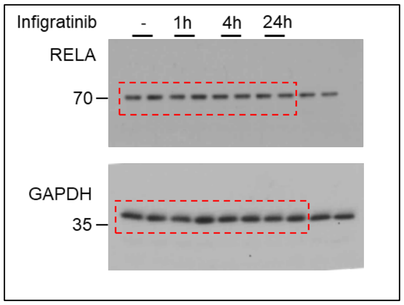


Fig S5e

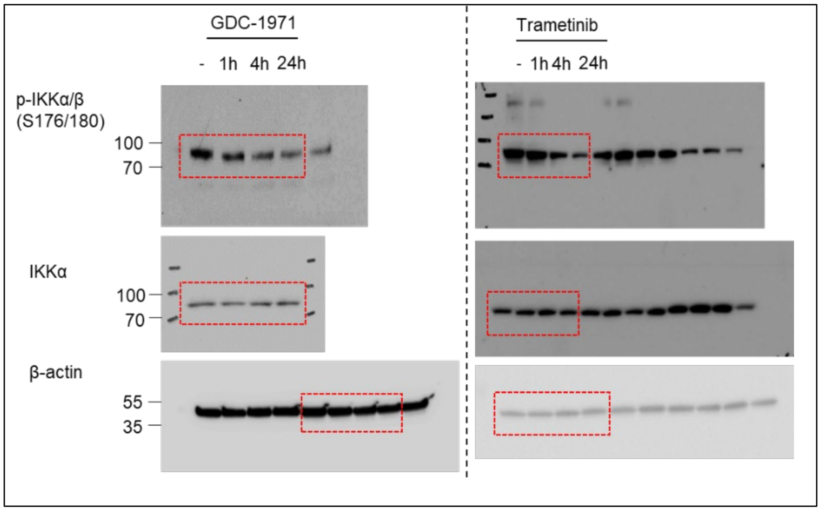

Source Data - Supplementary Figure 6

Fig S6a

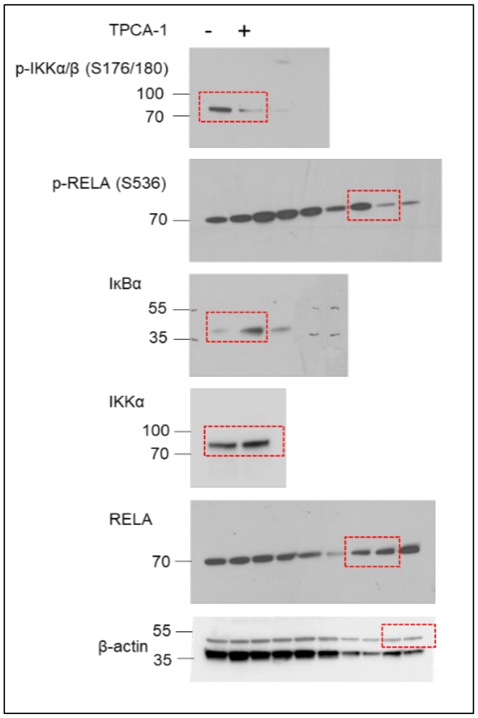


Fig S6b

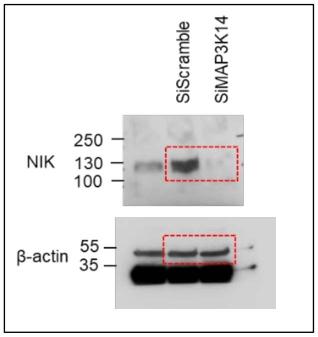


Fig S6e

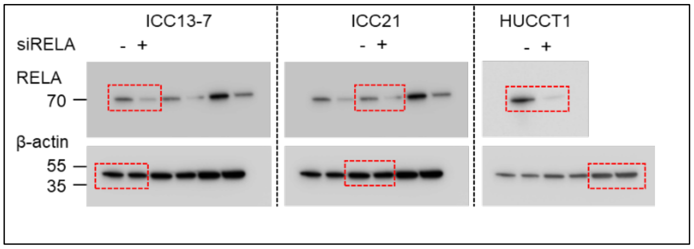

Source Data - Supplementary Figure 7

Fig S7i

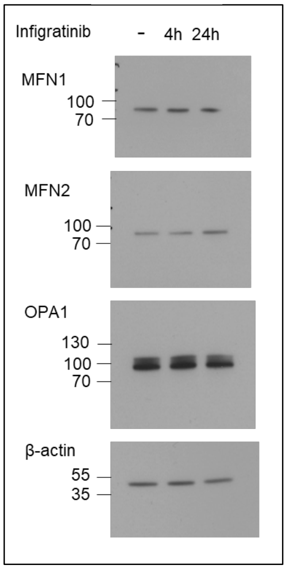


Fig S7l

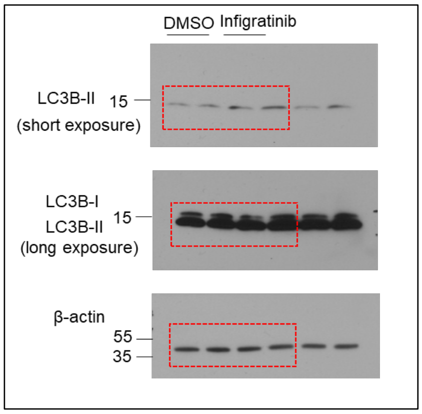


Fig S7m

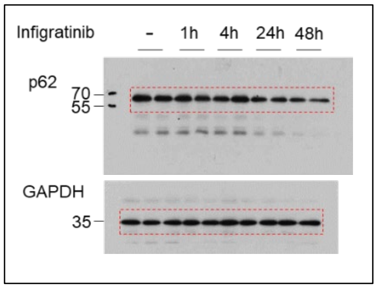

## Reference


1. Perera RM, Stoykova S, Nicolay BN, Ross KN, Fitamant J, Boukhali M, et al. Transcriptional control of autophagy–lysosome function drives pancreatic cancer metabolism. *Nature.* 2015;524(7565):361-5.
