# Supplementary material for: FGFR inhibition blocks NF-ĸB-dependent glucose metabolism and confers metabolic vulnerabilities in cholangiocarcinoma
Source: Nat Commun. 2024 May 7;15:3805. doi: 10.1038/s41467-024-47514-y (PMC11076599; doi:10.1038/s41467-024-47514-y)
Supplement: Supplementary file 3 — Reporting Summary [file 41467_2024_47514_MOESM3_ESM.pdf]

nature portfolio

Corresponding author(s): Nabeel Bardeesy

Last updated by author(s): 2024-2-4

Reporting Summary

Nature Portfolio wishes to improve the reproducibility of the work that we publish. This form provides structure for consistency and transparency in reporting. For further information on Nature Portfolio policies, see our Editorial Policies and the Editorial Policy Checklist.

Statistics

For all statistical analyses, confirm that the following items are present in the figure legend, table legend, main text, or Methods section.

n/a	Confirmed	
☐	☒	The exact sample size (*n*) for each experimental group/condition, given as a discrete number and unit of measurement
☐	☒	A statement on whether measurements were taken from distinct samples or whether the same sample was measured repeatedly
☐	☒	The statistical test(s) used AND whether they are one- or two-sided<br>*Only common tests should be described solely by name; describe more complex techniques in the Methods section.*
☒	☐	A description of all covariates tested
☒	☐	A description of any assumptions or corrections, such as tests of normality and adjustment for multiple comparisons
☐	☒	A full description of the statistical parameters including central tendency (e.g. means) or other basic estimates (e.g. regression coefficient) AND variation (e.g. standard deviation) or associated estimates of uncertainty (e.g. confidence intervals)
☐	☒	For null hypothesis testing, the test statistic (e.g. *F*, *t*, *r*) with confidence intervals, effect sizes, degrees of freedom and *P* value noted<br>*Give P values as exact values whenever suitable.*
☒	☐	For Bayesian analysis, information on the choice of priors and Markov chain Monte Carlo settings
☒	☐	For hierarchical and complex designs, identification of the appropriate level for tests and full reporting of outcomes
☒	☐	Estimates of effect sizes (e.g. Cohen's *d*, Pearson's *r*), indicating how they were calculated

Our web collection on statistics for biologists contains articles on many of the points above.

Software and code

Policy information about availability of computer code

Data collection	RNA seq: Trimmomatic v.0.36 was used to trim sequence reads to remove adapter sequences and nucleotides with poor quality. STAR aligner v.2.5.2b was used to map the trimmed reads to the Homo sapiens reference genome (ENSEMBL). DESeq2 was employed to compare expression between the sample. Metabolomics: MS1 data was analyzed using Compound Discoverer 3.3. Tracefinder was used to manually extract areas for compounds that could not be analyzed by Compound Discoverer.
Data analysis	Statistical Analysis: GraphPad Prism (https://www.graphpad.com); Transcriptional Analysis: Gene Set Enrichment Analysis (https://www.gsea-msigdb.org/gsea/index.jsp) and Enrichr (https://maayanlab.cloud/Enrichr/); Image quantification: Qupath (https://qupath.github.io); Seahorse analysis: Wave (https://www.agilent.com/en/product/cell-analysis/real-time-cell-metabolic-analysis/xf-software/seahorse-wave-desktop-software-740897); Phosphoproteomics Analysis: Kinase Library Platform (https://kinase-library.mit.edu/site); Metabolomics Analysis:MetaboAnalyst 6.0 (https://www.metaboanalyst.ca/MetaboAnalyst/ModuleView.xhtml); Schematics: Biorender (https://app.biorender.com)

For manuscripts utilizing custom algorithms or software that are central to the research but not yet described in published literature, software must be made available to editors and reviewers. We strongly encourage code deposition in a community repository (e.g. GitHub). See the Nature Portfolio guidelines for submitting code & software for further information.

Data

Policy information about availability of data

All manuscripts must include a data availability statement. This statement should provide the following information, where applicable:

- Accession codes, unique identifiers, or web links for publicly available datasets
- A description of any restrictions on data availability
- For clinical datasets or third party data, please ensure that the statement adheres to our policy

The RNA-Seq data reported in this study are deposited in the NCBI's Gene Expression Omnibus (GEO) database under accession code GSE241735 (https://www.ncbi.nlm.nih.gov/geo/query/acc.cgi?acc=GSE241735). Publicly available RNA-Seq data used in this study are from the ArrayExpress database at EMBL-EBI under accession number E-MTAB-11324 (https://www.ebi.ac.uk/biostudies/arrayexpress/studies/E-MTAB-11324?key=a8459a8a-c971-485c-9b53-0f94fc2a92ac). The metabolomics data reported in this study are deposited to Metabolomics Workbench (https://www.metabolomicsworkbench.org). The data can be accessed directly via its Project PR001775 (DOI: http://dx.doi.org/10.21228/M88M6V). All mass spectrometer raw files can be accessed through the MassIVE data repository (massive.ucsd.edu) under the accession number MSV000093916 (https://massive.ucsd.edu/ProteoSAFe/dataset.jsp?task=598187e239be46e3bfd7a26dc64a7a49). Source data are provided with this paper. All remaining data can be found in the Article, Supplementary or Source data files.

Research involving human participants, their data, or biological material

Policy information about studies with human participants or human data. See also policy information about sex, gender (identity/presentation), and sexual orientation and race, ethnicity and racism.

Reporting on sex and gender	The studies used patient-derived models of cholangiocarcinoma, from both male and female subjects.
Reporting on race, ethnicity, or other socially relevant groupings	N/A
Population characteristics	The patient-derived models were from both patients whose only treatment was surgical resection (MG69), who had been treated with chemotherapy and an FGFR inhibitor (ICC13-7, ICC21, ICC10-6), or treated with chemotherapy and immune checkpoint blockade (MG212)
Recruitment	N/A
Ethics oversight	MGH/DFCI

Note that full information on the approval of the study protocol must also be provided in the manuscript.

Field-specific reporting

Please select the one below that is the best fit for your research. If you are not sure, read the appropriate sections before making your selection.

[x] Life sciences [ ] Behavioural & social sciences [ ] Ecological, evolutionary & environmental sciences

For a reference copy of the document with all sections, see nature.com/documents/nr-reporting-summary-flat.pdf

Life sciences study design

All studies must disclose on these points even when the disclosure is negative.

Sample size	No sample-size calculation was performed. The sample sizes were chosen according to sample availability, sample variation, and previous experience in the field. The sample sizes were enough for the statistic analysis. Sample sizes were noted in the figure legends.
Data exclusions	No samples or animals were excluded from analysis
Replication	All attempts at replication generated reliable and similar results. The reproducibility of all the experiments is described in the figure legends.
Randomization	Mice were randomized in experiments assessing different treatments in vivo. Randomization was accomplished by preselecting mice at the time of initial compound administration for enrollment in each group. For in vitro experiments, randomization was not required as the samples are treated with defined conditions.
Blinding	Metabolomics, phosphoproteomics and RNAseq studies were conducted in a blinded manner. The other studies were not blinded since the majority of experiments were designed and performed by the same investigator, which made blinding not feasible.

Reporting for specific materials, systems and methods

We require information from authors about some types of materials, experimental systems and methods used in many studies. Here, indicate whether each material, system or method listed is relevant to your study. If you are not sure if a list item applies to your research, read the appropriate section before selecting a response.

Materials & experimental systems

n/a	Involved in the study
☐	☒ Antibodies
☐	☒ Eukaryotic cell lines
☒	☐ Palaeontology and archaeology
☐	☒ Animals and other organisms
☒	☐ Clinical data
☒	☐ Dual use research of concern
☒	☐ Plants

Methods

n/a	Involved in the study
☒	☐ ChIP-seq
☐	☒ Flow cytometry
☒	☐ MRI-based neuroimaging

Antibodies

Antibodies used

The primary antibodies used in the study included (The application is for Western blot unless specifically mentioned): from cell signaling technology, p-FRS2 (Y196) (3864), p-ERK1/2 (T202/Y204) (4370, clone D13.14.4E), ERK1/2 (4695, clone 137F5), p-RELA/p65 (S536) (3031), RELA/p65 (8242, clone D14E12), p-IKKα/β (S176/180) (2697, clone 16A6), IKKα (11930, clone 3G12), IkBα (4812, clone 44D4), NIK (4994), NF-kB2/p100/p52 (37359, clone D7A9K), β-Tubulin (86298, clone D3U1W), PARP (9542), HK2 (2867, clone C64G5), LDHA (3582, clone C4B5), PKM2 (4053, clone D78A4), p-DRP1 (S616) (3455), DRP1 (8570, clone D6C7), MFN1 (14739, clone D6E2S), MFN2 (9482, clone D2D10), OPA1 (80471, clone D6U6N), p62 (23214, clone D6M5X), LC3B (3868, clone D11) for immunoblot with dilution 1:1,000-1:3,000; from Millipore-Sigma, β-actin (A5316, clone AC-74) with dilution 1:20,000; from Abcam, FRS2 (ab183492), CLPX (ab168338, clone EP8772) with dilution 1:1,000; from Servicebio for immunofluorescence, KI67 (GB13030-2, 1:200); from Novus Biologicals for immunofluorescence, pan-cytokeratin (NBP2-29429, clone AE-1/AE-3, 1:200); from MyBioSource, GLUT1 (MBS9126610, 1:2,000); from MBL, LC3B (PM036, 1:200) for immunofluorescence. The secondary antibodies used in the study included: horseradish peroxidase (HRP)-conjugated secondary antibodies (Vector Laboratories: anti-rabbit, PI-1000; anti-mouse, PI-2000) with dilution 1:10,000.

Validation

RELA/p65, NIK, and NF-kB2 (p100/p52) antibodies were validated in human samples for Western blot by gene knockdown. Validation of other commercial antibodies is available in the product page and search of relevant literature. See following.
Western blot:
p-FRS2 (Y196) (3864): https://www.cellsignal.com/products/primary-antibodies/phospho-frs2-a-tyr196-antibody/3864
p-ERK1/2 (T202/Y204) (4370, clone D13.14.4E):https://www.cellsignal.com/products/primary-antibodies/phospho-p44-42-mapk-erk1-2-thr202-tyr204-d13-14-4e-xp-174-rabbit-mab/4370
ERK1/2 (4695, clone 137F5):https://www.cellsignal.com/products/primary-antibodies/p44-42-mapk-erk1-2-137f5-rabbit-mab/4695
p-RELA/p65 (S536) (3031):https://www.cellsignal.com/products/primary-antibodies/phospho-nf-kb-p65-ser536-antibody/3031
RELA/p65 (8242, clone D14E12):https://www.cellsignal.com/products/primary-antibodies/nf-kb-p65-d14e12-xp-174-rabbit-mab/8242
p-IKKα/β (S176/180) (2697, clone 16A6):https://www.cellsignal.com/products/primary-antibodies/phospho-ikka-b-ser176-180-16a6-rabbit-mab/2697
IKKα (11930, clone 3G12):https://www.cellsignal.com/products/primary-antibodies/ikka-3g12-mouse-mab/11930
IkBα (4812, clone 44D4):https://www.cellsignal.com/products/primary-antibodies/ikba-44d4-rabbit-mab/4812
NIK (4994):https://www.cellsignal.com/products/primary-antibodies/nik-antibody/4994
NF-kB2/p100/p52 (37359, clone D7A9K):https://www.cellsignal.com/products/primary-antibodies/nf-kb2-p100-p52-d7a9k-rabbit-mab/37359
β-Tubulin (86298, clone D3U1W):https://www.cellsignal.com/products/primary-antibodies/b-tubulin-d3u1w-mouse-mab/86298
PARP (9542):https://www.cellsignal.com/products/primary-antibodies/parp-antibody/9542
HK2 (2867, clone C64G5):https://www.cellsignal.com/products/primary-antibodies/hexokinase-ii-c64g5-rabbit-mab/2867
LDHA (3582, clone C4B5):https://www.cellsignal.com/products/primary-antibodies/ldha-c4b5-rabbit-mab/3582
PKM2 (4053, clone D78A4):https://www.cellsignal.com/products/primary-antibodies/pkm2-d78a4-xp-rabbit-mab/4053
p-DRP1 (S616) (3455):https://www.cellsignal.com/products/primary-antibodies/phospho-drp1-ser616-antibody/3455
DRP1 (8570, clone D6C7):https://www.cellsignal.com/products/primary-antibodies/drp1-d6c7-rabbit-mab/8570
MFN1 (14739, clone D6E2S):https://www.cellsignal.com/products/primary-antibodies/mitofusin-1-d6e2s-rabbit-mab/14739
MFN2 (9482, clone D2D10):https://www.cellsignal.com/products/primary-antibodies/mitofusin-2-d2d10-rabbit-mab/9482
OPA1 (80471, clone D6U6N):https://www.cellsignal.com/products/primary-antibodies/opa1-d6u6n-rabbit-mab/80471
p62 (23214, clone D6M5X):https://www.cellsignal.com/products/primary-antibodies/sqstm1-p62-d6m5x-rabbit-mab/23214
LC3B (3868, clone D11): https://www.cellsignal.com/products/primary-antibodies/lc3b-d11-xp-174-rabbit-mab/3868
β-actin (A5316, clone AC-74): https://www.sigmaaldrich.com/US/en/product/sigma/a5316
FRS2 (ab183492):https://www.abcam.com/products/primary-antibodies/frs2-antibody-epr14724-ab183492.html
CLPX (ab168338, clone EP8772):https://www.abcam.com/products/primary-antibodies/clpx-antibody-ep8772-ab168338.html
GLUT1 (MBS9126610):https://www.mybiosource.com/antibody/slc2a1/9126610

Immunofluorescence:
KI67 (GB13030-2):https://www.citeab.com/antibodies/10504489-gb13030-2-anti-ki67-rabbit-mab; and our previous publications (PMID: 35420673 and PMID: 34848557)
pan-cytokeratin (NBP2-29429, clone AE-1/AE-3):https://www.novusbio.com/products/cytokeratin-pan-antibody-ae-1-ae-3_nbp2-29429
LC3B (PM036):https://www.mblbio.com/bio/g/dtl/A/?pcd=PM036

Eukaryotic cell lines

Policy information about cell lines and Sex and Gender in Research

Cell line source(s)	The following models were generated from patients treated at Massachusetts General Hospital and established under an IRB-approved protocol, including the cholangiocarcinoma cell lines, ICC13-7 (female), ICC10-6 (Male), and ICC21 (Female), ICC12, ICC10-8 and patient-derived xenografts of cholangiocarcinoma, MG69 (male) and MG212 (female).<br><br>HuCCT1 cells were obtained from Riken Bioresource Center.CC-SW-1 and SG231 were from Dr. Theresa L. Whiteside (University of Pittsburgh).
Authentication	STR fingerprinting was done at ATCC and the Broad Institute. STR profiles were compared with STR profiles reported by ATCC and in the literature. The STR information is available on DepMap portal, or from the authors upon reasonable request.
Mycoplasma contamination	All cell lines were regularly tested for mycoplasma contamination uses a commercial kits as noted in the Methods, and were negative in all cases.
Commonly misidentified lines (See ICLAC register)	No commonly misidentified lines were used.

Animals and other research organisms

Policy information about studies involving animals; ARRIVE guidelines recommended for reporting animal research, and Sex and Gender in Research

Laboratory animals	Mice were maintained with standard protocols for laboratory animal care. Mice were housed in controlled environments to ensure their well-being and to maintain experimental consistency. The housing conditions adhered to established guidelines outlined in the Institutional Animal Care and Use Committee (IACUC) regulations (Protocol 2005N000148 and 2019N000116). Mice were monitored daily by a staff with dedicated expertise in research animal welfare.<br>The mice were housed in polycarbonate cages allowing 5 mice per cage in a dedicated animal facility at the Simches Research Building. Bedding material consisting of autoclaved wood shavings, and was changed regularly, The environment had controlled temperature (20-24°C) and humidity (40-60%) levels. Lighting conditions followed a standard 12-hour diurnal cycle. Enrichment was provided in the form of nesting material. Mice were given ad libitum access to standard laboratory rodent chow (Prolab® IsoPro® RMH 3000, 5P75-RHI-W 23). Water was provided through an automatic watering system.<br>For subcutaneous tumor studies, 3x10^6 ICC13-7 cells (in 50% matrigel) were injected subcutaneously into the lower flank of NOD-scid IL2R-gamma-null mice (6-10 weeks of age) from Jackson Laboratories, strain #005557. Tumor size was assessed at indicated time points by caliper measurements of length and width and the volume was calculated according to the formula ([length × width2]/2). Tumor growth was followed until the animals reached humane endpoints.
Wild animals	No wild animals were used.
Reporting on sex	In vitro cell lines were derived from human patients of both sexes.<br>For in vivo studies, we matched the sex of the recipient animal with that of the subject from whom the model was derived.<br>We acknowledge that sex and gender may be important confounding factors in some experimental settings. For the experiments in the present study, however, we are not aware of significant differences that may arise when segregating by sex and do not believe that the results apply to only one sex or gender. We did not collect data separately by sex (or gender), consistent with previous studies, and therefore are unable to provide disaggregated data at this time. We understand that sex may be a significant variable

	and will seek to incorporate sex and gender into future study designs.
Field-collected samples	No field-collected samples.
Ethics oversight	All mice experiments were conducted under protocols 2005N000148 and 2019N000116 approved by the Institutional Animal Care and Use Committee at Massachusetts General Hospital and comply with all regulations for the ethical conduct of research. The MGH Institutional Animal Care and Use Committee regulations for maximum tumor size (<2 cm in greatest diameter) were strictly adhered to.

Note that full information on the approval of the study protocol must also be provided in the manuscript.

Flow Cytometry

Plots

Confirm that:

- [x] The axis labels state the marker and fluorochrome used (e.g. CD4-FITC).
- [x] The axis scales are clearly visible. Include numbers along axes only for bottom left plot of group (a 'group' is an analysis of identical markers).
- [x] All plots are contour plots with outliers or pseudocolor plots.
- [x] A numerical value for number of cells or percentage (with statistics) is provided.

Methodology

Sample preparation	For LC-3 analysis: ICC13-7 cells were engineered to stably express the LC3-GFP-mCherry construct. Then cells were subjected to DMSO or Infigratinib treatment for 48 hours. After treatment, cells were digested and resuspended in culture media.
Instrument	Samples were run on a Cytoflex S flow cytometer (Beckman Coulter Life Sciences).
Software	Data were analyzed using FlowJo (Tree Star, Ashland, OR) software.
Cell population abundance	At least 10,000 cells were analyzed per condition
Gating strategy	Gates and regions are placed around populations of cells with defined characteristics. The singlets were gated out based on FSC/SSC. LC3-mcherry positive cells were gated out based on PE values for further analysis (ratio of PE to FITC).

- [x] Tick this box to confirm that a figure exemplifying the gating strategy is provided in the Supplementary Information.
